# Real-time audiovisual feedback system in a physician-staffed helicopter emergency medical service in Finland: the quality results and barriers to implementation

**DOI:** 10.1186/1757-7241-21-50

**Published:** 2013-07-01

**Authors:** Marko Sainio, Antti Kämäräinen, Heini Huhtala, Petri Aaltonen, Jyrki Tenhunen, Klaus T Olkkola, Sanna Hoppu

**Affiliations:** 1 Department of Intensive Care Medicine, Tampere University Hospital and University of Tampere, PO Box 2000, Tampere, FI-33521, Finland; 2 Department of Emergency Medicine, Tampere University Hospital and University of Tampere, PO Box 2000, Tampere, FI-33521, Finland; 3 School of Health Sciences, University of Tampere, Tampere, FI-33014, Finland; 4 Department of Anaesthesiology, Intensive Care, Emergency Care and Pain Medicine, University of Turku and Turku University Hospital, PO Box 52 (Kiinamyllynkatu 4–8), Turku, FI-20521, Finland; 5 Department of Surgical Sciences, Anaesthesiology and Intensive Care, Uppsala University, Uppsala, SE 751 85, Sweden

**Keywords:** CPR, Quality, Resuscitation, Cardiac arrest, Pre-hospital, HEMS

## Abstract

**Objectives:**

To evaluate the quality of cardiopulmonary resuscitation (CPR) in a physician staffed helicopter emergency medical service (HEMS) using a monitor-defibrillator with a quality analysis feature. As a post hoc analysis, the potential barriers to implementation were surveyed.

**Methods:**

The quality of CPR performed by the HEMS from November 2008 to April 2010 was analysed. To evaluate the implementation rate of quality analysis, the HEMS database was screened for all cardiac arrest missions during the study period. As a consequence of the observed low implementation rate, a survey was sent to physicians working in the HEMS to evaluate the possible reasons for not utilizing the automated quality analysis feature.

**Results:**

During the study period, the quality analysis was used for 52 out of 187 patients (28%). In these cases the mean compression depth was < 40 mm in 46% and < 50 mm in 96% of the 1-min analysis intervals, but otherwise CPR quality corresponded with the 2005 resuscitation guidelines. In particular, the no-flow fraction was remarkably low 0.10 (0.07, 0.16). The most common reasons for not using quality-controlled CPR were that the device itself was not taken to the scene, or not applied to the patient, because another EMS unit was already treating the patient with another defibrillator.

**Conclusions:**

When quality-controlled CPR technology was used, the indicators of good quality CPR as described in the 2005 resuscitation guidelines were mostly achieved albeit with sufficient compression depth. The use of the well-described technology in improving patient care was low. Wider implementation of the automated quality control and feedback feature in defibrillators could further improve the quality of CPR on the field.

**Trial registration:**

ClinicalTrials.gov (NCT00951704)

## Introduction

The quality of cardiopulmonary resuscitation (CPR), as quantified by CPR quality variables such as chest compression depth, compression rate, full chest recoil and minimal interruptions in CPR are correlated with the likelihood of return of spontaneous circulation (ROSC) after cardiac arrest [1-5]. This is plausible since low-quality CPR has been shown to be associated with several negative haemodynamic effects [6-8]. Although we are aware of the importance of CPR quality to survival after cardiac arrest, recent reports have described significant deficiencies in the quality of CPR provided by health care personnel both in hospital [9] and pre-hospital environment [10].

Modern defibrillator technology enables continuous monitoring of CPR quality using a sternal accelerometer/force transducer and impedance changes across the defibrillation electrodes which facilitates automated real-time feedback. Although the use of a prototype of such a defibrillator has increased the quality of CPR during prehospital resuscitation [11] this technology is not routinely used. An additional potential benefit for the quality of CPR comes through the possibility for post-event debriefing [12,13].

The purpose of this study was to evaluate CPR quality in a physician staffed helicopter emergency medical service (HEMS) in Southwest Finland. We also analysed the implementation rate of real- time audio-visual feedback device in CPR and tried to ascertain why it was not used in all resuscitation attempts.

## Material and methods

The data on pre-hospital cardiac arrests treated by the HEMS of the Turku area in Southern Finland were collected prospectively between 1 November 2008 and 30 April 2010. The HEMS serves approximately 630 000 inhabitants including the city of Turku with a population of 175.000. The majority of the HEMS physicians are specialists in anaesthesiology and intensive care medicine. In addition to the HEMS, the EMS system includes first responding units (FRU) and basic life support (BLS) units staffed with firemen-emergency medical technicians (EMT) and paramedic staffed advanced life support (ALS) units. Alongside a FRU and the nearest BLS or ALS unit, the HEMS is always dispatched to high-risk medical emergencies such as suspected cardiac arrest. In most emergencies the HEMS usually arrives few minutes after the land crew. Successfully resuscitated patients with return of spontaneous circulation (ROSC) are transported to the Turku University Hospital, which is a tertiary referral centre providing therapeutic hypothermia and percutaneous coronary intervention for cardiac arrest victims when indicated.

The study protocol was approved by the Institutional Review Board of the Tampere University Hospital and registered in clinicaltrials.gov (NCT00951704). All patients ≥ 18 years of age suffering from sudden prehospital cardiac arrest regardless of the initial rhythm or presumed aetiology were considered eligible to the study. The ethics committee waived the written informed consent procedure due to the observational nature of the study and because we were including only adult patients.

In addition to common emergency medical equipment, the HEMS unit was equipped with a HeartStart MRx defibrillator with Q-CPR™ option, jointly designed by Philips Health Care (Andover, MA, USA) and Laerdal Medical AS (Stavanger, Norway). All physicians received extra training in the use of the real time audio-visual feedback device (Q-CPR) with repeated instructions and reminders to use of the quality analysis feature. During the study period, resuscitation followed the 2005 ILCOR guidelines [14].

The physicians were trained to ensure that the oval shaped compression sensor (sternal accelerometer/force transducer) was attached to the patient’s chest at the scene and CPR was to be continued according to the feedback from the Q-CPR. Attaching the sensor on the chest is quick and has no impact on hands-off time (time without chest compression). Furthermore one can assume that it does not confound the teamwork needed during CPR. However, the decision whether or not to use the Q-CPR feature during resuscitation attempts was left at the physician’s discretion.

During each resuscitation the device recorded compression-to-compression depth, compression rate, compression-to-decompression duty cycle, incomplete chest compression release, hands-off (no-flow) time, pre- and post-shock pauses and ventilation rate. As indicators of quality, compression depth, rate and release and ventilation rate were monitored during CPR with automated real-time audiovisual feedback. During the study period CPR goals were a compression rate of 90 to 120/min, depth of 38 to 51 mm and a ventilation rate of 8 to 12/min.

The detailed information on the performance during the resuscitation were processed and analysed with Q-CPR Review software v2.1.1.0. (Laerdal Medical AS, Stavanger, Norway). Uniform guidelines for reporting CPR quality established by an international consensus working-group were used [15].

In the primary analysis each resuscitation attempt was divided into 1-min intervals for evaluation, and measured variables included overall average compression depth (mm), compression rate (CC/min), and compression count (actual number of compressions delivered per minute), no flow time (NFT, time without chest compressions) per 1-min intervals, likewise the no flow fraction (NFF, the fraction of time with no compressions during cardiac arrest). Further, percentages of intervals with an average depth < 40 mm, < 50 mm or average rates < 90 or > 120 min^-1^ were also determined. Incomplete chest release percentage was calculated from compressions delivered per minute, from total number of the episode compressions and from total number of all compressions. Pre- and post-shock pauses (s) were analysed. Restoration of spontaneous circulation (ROSC), 24-h survival, survival to hospital discharge and neurological status six months after discharge were also analysed. In addition, we calculated the number of cases where the Q-CPR feature was used.

In the secondary analysis we retrieved the data on all cardiac arrests (resuscitation attempts with or without the real time audio-visual feedback feature) and assessed the basic characteristics and outcome details according to the Utstein style [16]. Neurological status at the discharge was assessed by reviewing of the medical records and classified by degree of reported disability and discharge destination.

The descriptive survey was done by questionnaire to all HEMS physicians and the aim was to assess the reasons for the low rate of Q-CPR feature use. The questionnaire included the following questions: 1. “Have you attended resuscitation scenarios while working in the HEMS?” A: Yes or No, 2. “Was the Q-CPR feature used in all cases?” A: Yes or No, 3. “If the Q-CPR feature was not used, please specify the reason.” A: a. “Q-CPR device was not carry to the scene”, b) “device was not used, because another EMS unit was already treating the patient with another defibrillator”, c) “it is unnecessary to use the real time audio-visual feedback feature in every cardiac arrest patient”, d) “another reason, what?”

Statistical analyses were performed with SPSS for windows (SPSS version 19.0, Chicago, IL). Data are presented as numbers and percentages of patients, as median with interquartile range (IQR), or as means and standard deviations (SD) pending the normality of distribution. Differences between groups were analysed using the *χ*[2]-test with continuity correction for categorical data and Student’s *t* test or Mann–Whitney *U* test for continuous data as appropriate.

## Results

During the 18-month study period the HEMS accomplished a total of 5,318 missions. These included 391 cases of cardiac arrest in which either the FRU or the HEMS considered resuscitation. In 338 cases (86%) the FRU initiated resuscitation at the scene. In 46 (12%) cases the paramedics considered the attempt futile and the HEMS physicians confirmed this. One patient had a do-not-attempt-resuscitation (DNAR) order and in six cases the data were missing.

**Figure 1:**
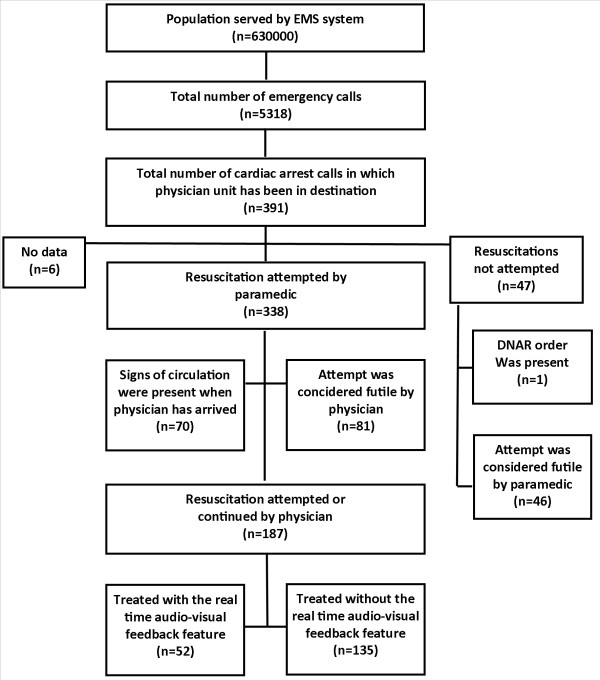
The Utstein style flow chart of core data elements.

At the time of HEMS arrival, the FRU or the paramedics had achieved ROSC in 70 patients. In 81 cases the HEMS physician considered further resuscitation attempts futile. In the final analysis, out of the initial 391 cardiac arrests there were 187 (48%) patients on the HEMS initiated or continued resuscitation. The Utstein style flow chart of core data elements is presented in Figure 1.

Of the 187 patients, quality-controlled CPR was provided in 52 cases (28%). The median duration of the resuscitation with real-time audio-visual feedback feature was 12 min 6 s (7 min 48 s, 16 min 53 s). The mean compression depth was 40 (6) mm and in 46% out of the 1-min intervals compression depth remained < 40 mm. In 20 of the 52 resuscitation attempts the mean compression depth was sufficient (>40 mm) in >60% of the 1-min intervals. The mean compression rate was 108 (10) min^-1^, with 10% of all 1-min intervals with a compression rate < 90 or > 120 min^-1^. The median no-flow fraction was 0.10 (0.07, 0.16) and the mean no-flow time per 1-min fraction was 7 (9) seconds, resulting in actual compressions delivered per minute at a mean rate of 95 (20) min^-1^. The median incomplete chest release percentage out of the total number of compressions was 3 (1, 14)%, and the median pre- and post-shock pauses were 9 (5, 15) and 3 (2, 5) s respectively (Table 1).

**Table 1:** Data on quality of cardiopulmonary resuscitation (CPR) treated by physicians with Q-CPR (n = 52)

Episode duration, median (IQR), min:s	12:06 (7:48, 16:53)
Total number of compressions, (No.)	62895
Compression depth (mm)	
Episode mean (SD)	40 (6)
Fraction of 1-min epochs with compression depth < 40 mm, No. (%)	292/629 (46)
Fraction of 1-min epochs with compression depth < 50 mm, No. (%)	604/629 (96)
Compression depth within recommended (>40 mm) > 60% of episodes 1-min epochs, No (%)	20/52 (39)
Compression rate (min^-1^)^a^	
Episode mean (SD)	108 (10)
Fraction of minutes with compression rate <90 or >120 min^−1^, No. (%)	60/629 (10)
Compressions delivered (min^-1^), episode mean (SD)^b^	95 (20)
Compressions with incomplete release^c^	
Number of total compressions, No. (%)	5158/62895 (8)
Percentage of total number of episode compressions, median (IQR)	3 (1, 14)
Incomplete chest release per 1 min segments, median (IQR),%	0 (0, 6)
Compression as part of duty cycle, episode mean (SD),%	43 (4)
No flow time (pauses)^d^	
No-flow fraction, median (IQR)	0.10 (0.07, 0.16)
No flow time per 60 s fraction, mean (SD), s	7 (9)
Pre-shock pause, median (IQR)^e^, s	9 (5, 15)
Post-shock pause, median (IQR)^f^, s	3 (2, 5)

The data are presented as numbers and percentages of patients, as median and 25% to 75% interquartile range (IQR), or as means and standard deviations (SD) as appropriate (non-normal or normal distribution respectively).

^a^Indicates the rate of compressions when delivered.

^b^Indicates the average number of compressions actually given per minute during the resuscitation attempt.

^c^Indicates the incomplete chest wall decompression after chest compression phase.

^d^Indicates the proportion of time without chest compressions during the resuscitation attempt.

^e^Indicates the proportion of time during rhythm analyses? without chest compressions before shock.

^f^Indicates the proportion of time during rhythm analyses? without chest compressions after shock.

In the secondary analysis the median HEMS unit response interval to cardiac arrest patients treated with or without the real time audio-visual feedback feature was 12 (10, 15) and 12 (10, 15) min (p = NS) respectively. The median delays from HEMS arrival to ROSC achieved with or without the real time audio-visual feedback feature were 12 (10, 15) and 7 (5, 17) min (p = 0.05) respectively. Demographic and resuscitation episode characteristics and outcome parameters of the two groups are presented in Table 2.

**Table 2:** Demographics and quality of cardiopulmonary resuscitation (CPR) treated by physicians

**Characteristic**	**With Q-CPR (n = 52)**	**Without Q-CPR (n = 135)**	p-Value^*^
Age, mean (SD), y	66 (17)	60 (20)	0.20
Data not available	0	5	
Male sex, No. (%)	35 (67)	97 (72)	0.54
Cardiac aetiology, No. (%)	42 (81)	89 (66)	0.05
Location of arrest, No. (%)			
Home	23 (44)	74 (55)	0.19
Public	21 (40)	34 (25)	0.04
Other	8 (15)	26 (19)	0.54
Data not available	0	1	
Cardiac arrest witnessed status, No. (%)			
Bystander witnessed	30 (58)	87 (64)	0.39
EMS personnel witnessed	9 (17)	23 (17)	0.97
Arrest not witnessed	13 (25)	25 (19)	0.32
FRU unit:			
Response interval in all cases, median (IQR), min	5 (2, 8)	6 (2, 10)	0.68
Data not available	3	19	
Response interval in VF/VT cases, median (IQR), min	6 (4, 10)	7 (3, 10)	0.73
Data not available	1	2	
HEMS unit:			
Responded with helicopter, No. (%)	13 (25)	45 (33)	0.23
Response interval (all), median (IQR), min	12 (10, 15)	12 (10, 15)	0.58
Data not available (%)	3 (6)	2 (1)	
Response interval of helicopter, median (IQR), min	12 (10, 16)	17 (12, 24)	0.22
Response interval of rapid response vehicle, median (IQR), min	12 (10, 15)	11 (9, 13)	0.49
Initial rhythm, No. (%)			
VF/VT	22 (42)	38 (28)	0.06
Asystole	12 (23)	50 (37)	0.07
PEA	18 (35)	44 (33)	0.79
Data not available	0	3	
Time from call to ROSC, median (IQR), min	26 (21, 29)	24 (15, 29)	0.24
Time from HEMS unit arrival to ROSC, median (IQR), min	12 (10, 15)	7 (5, 17)	0.05
Patient outcome, No. (%)			
Any ROSC	28 (54)	49 (36)	0.03
Primary survival to ED/ICU	22 (42)	35 (26)	0.03
Alive after 24 hours	18 (35)	29 (22)	0.06
Discharged from hospital alive	8 (15)	22 (16)	0.88
Data not available	0	2	
Neurological outcome at 6 month all patient, No. (%)			
CPC 1-2	8 (15)	13 (10)	0.26
CPC 3	0	0	
CPC 4-5	44 (85)	113 (84)	0.87
Data not available	0	9	

Abbreviations: *FRU* First responding units, *HEMS* Helicopter emergency medical service, VF, ventricular fibrillation, *VT* Ventricular tachycardia, *PEA* Pulseless electrical activity, *ROSC* Return of spontaneous circulation, *ED*, Emergency department, *ICU*, Intensive care unit, *CPC*, Cerebral performance category.

The data are presented as numbers and percentages of patients, as median and 25% to 75% interquartile range (IQR) or as mean and standard deviation (SD) as appropriate (non-normal or normal distribution respectively).

^*^The differences between groups were analysed using chi-square test with continuity correction for categorical data and *t* test or Mann–Whitney test for continuous data as appropriate. No trauma related cardiac arrest patients were enrolled as by chance.

### Survey

Eight HEMS physicians out of 11 responded to the survey. Six respondents were specialists in anaesthesiology and intensive care medicine, one an attending internist and one a senior resident in anaesthesiology and intensive care. All respondents had attended resuscitation attempts during the study period and used the real time audio-visual feedback feature during resuscitation attempts. All respondents had also attended resuscitation attempts in which the Q-CPR had not been used. The most common reason for not using quality-controlled CPR was failure to carry the Q-CPR device to the scene. Secondly, another EMS unit was in many cases already treating the patient with another defibrillator. All physicians considered it unnecessary to use the real-time audio-visual feedback feature on every cardiac arrest patient, especially if the response time had been long.

## Discussion

In this study HEMS physicians used the real time audio-visual feedback feature in only 28% of all resuscitation attempts. When asked, HEMS physicians reported three main reasons for not using the real time audio-visual feedback feature to guide resuscitation in all resuscitation attempts. First, when a primary responding unit had already initiated resuscitation using a defibrillator without the quality analysis feature, it was generally considered unnecessarily burdensome or useless to change the equipment. Second, in some cases the defibrillator with the quality analysis feature had not been brought to the patient at all and third, in few cases the use of the Q-CPR was not considered worthwhile when the arrival of the HEMS was delayed. Regarding the time delays, the median HEMS response interval in all patients treated without Q-CPR was only 12 min in our study, which contradicts the assumption that long response intervals contraindicated the use of the real-time audio-visual feedback feature. The responses to the survey exemplify attitudes that need to be tackled through training and research.

Recent studies report that real-time feedback devices used during a resuscitation attempt can modify CPR performance and improve adherence to resuscitation guidelines [11,17-22]. This *per se* is an important goal. Although the main aim in using real time audio-visual feedback is to increase the likelihood of performing CPR according to the international resuscitation guidelines, in this study only 20 cases (38%) had the mean compression depth within the recommendation of more than 60% of the 1-min intervals. In the latest 2010 resuscitation guidelines the target compression depth is at least 50 mm [23]. Interestingly, the mean compression depth remained < 50 mm in 96% of all 1-min intervals in this study. The other quality variables such as mean compression rate, compressions delivered min^-1^, incomplete chest release and no-flow fraction were mostly within the recommended range. Of note, no flow fraction was very low 0.10 (0.07, 0.16). Two recently published prospective observational cohort studies demonstrated relationship between compression fraction (1- no-flow fraction) and likelihood of ROSC in OCHA patients with confirmed ventricular fibrillation and also patients with not in ventricular fibrillation. High compression fraction was independently predictive of better survival in patients who experience a prehospital ventricular fibrillation/tachycardia cardiac arrest [3,4]. However, there is little if any data demonstrating that quality controlled CPR actually improves patient outcome in out of hospital setting [21,24,25]. This may be reflected in emergency physicians’ attitudes and result in low utilisation of the quality analysis feature. A recently published report by the Resuscitation Outcomes Consortium (ROC) investigators evaluated whether real-time feedback increases the likelihood of ROSC in out-of-hospital setting. The use of real time audio-visual feedback improved the mean compression depth by only 2 mm (from 37.7 mm to 39.7 mm) and the fraction of the time with active chest compression from 64% to 66%. The investigators therefore concluded that likelihood of ROSC was not associated with the use real time audio-visual feedback [25]. Stiell et al. demonstrated recently an association between survival and increased compression depth in pre-hospital setting, but also numerous deficiencies in achieving recommended chest compression depth despite having real time audio-visual feedback [5], which concurs with our small-scale study. These studies demonstrate the need to measure chest compression depth routinely during CPR, and it should be noted that real time audio-visual feedback does not by itself guarantee sufficient chest compression depth or low no-flow fraction, and future studies are needed to analyze the impact of the nontechnical skills to the quality of resuscitation attempt.

Studies have shown a delay of over a year in the implementation of resuscitation guidelines in clinical practice [26,27]. Bigham et al. interviewed 176 EMS agencies to elicit “what issue, if any, delayed implementation of the guidelines into field practice”. In that survey the three main barriers were insufficient training, technical aspects and reluctance to accept new guidelines in clinical practice [28]. A study from Scotland reported that real-time feedback with targeted training improves the quality of pre-hospital resuscitation. Unfortunately, it was underpowered to demonstrate an association, or lack thereof, between improved CPR quality and better outcome [22]. During our study period of 18 months the use of the Q-CPR was rarely deployed despite initial training and repeated instructions and reminders. One explanation could be that the HEMS physicians received no feedback on the quality of the resuscitation attempts during the study period. It has previously been observed that post-event reporting and debriefing have a positive impact on the quality of resuscitation [17,29].

To improve CPR quality by deploying CPR feedback/prompt devices, it is crucial for emergency physicians and other professionals to understand the operating principle of the equipment. Demonstrating that using Q-CPR technology improves survival will make such technology more readily accepted.

This study has limitations. First, although the initial data collection on the use of Q-CPR was prospective, the final survey on the success rate of the implementation was retrospective and post hoc in nature. Second, due to the low implementation rate the number of cases in which Q-CPR was used remained low. However, the latter limitation seems to reflect the real life obstacle observed in this study, which *per se* could not be prospectively addressed without risking the Hawthorne effect [30]. Finally, the reader is reminded to bear in mind the low number of patients, observational non-randomized design and in particular the primary rhythm differences between the groups when considering the relevance of the outcome measures in this report. Our primary aim was not to report outcome measurers for the between the groups comparison, rather to describe the overall patient cohort and performance.

The survey responses represent respondents’ subjective perceptions in general and the reasons why not using the Q-CPR feature cannot be defined in detail. There may have been cases where the clinical or operative situation did not permit the use of quality analysis, but the study setting allows no in-depth analysis of these situations.

## Conclusions

In the present study, HEMS physicians made little use of a well-described technology with potential for improving patient care. When quality-controlled CPR technology was used, the indicators of good quality CPR were mostly achieved, but compression depth was insufficient. If we want to improve CPR quality via implementation of CPR feedback/prompt devices, it is of utmost importance that HEMS physicians and other professionals understand the operational principle of the equipment. If we can prove that the use Q-CPR technology improves survival, it is not difficult to increase the popularity of such technology. Knowledge, training and understanding the purpose of new technology are key elements for successful implementation of new devices and protocols such as quality analysis and feedback during CPR. Further evidence to support the potential benefits of quality analysis during CPR, extensive training of medical professionals and a wider availability of defibrillators with the quality analysis feature could be the means to achieve improved resuscitation quality.

## Competing interests

Dr. Jyrki Tenhunen has been a member of international advisory board for SuPARnostic (Virogates, Copenhagen, Denmark) and is CMO and shareholder in SenSem Technologies Ltd (Tampere, Finland.) and Medieta Ltd (Helsinki, Finland). Dr. Sanna Hoppu has provided paid consultancy for Laerdal Medical Corporation. Laerdal Medical has not funded any part of this study, nor had access to any of the data or partaken in the process of the study. Other authors have no conflicts of interest to declare.

## Authors’ contributions

MS had full access to all of the data in the study and takes responsibility for the integrity of the data and the accuracy of the data analysis. Study concept and design: MS, HS, PA. Critical revision of the manuscript for important intellectual content: MS, AK, JT, KTO, SH. Statistical analysis: MS. HH. Analysis and interpretation of data: MS, AK, JT, KTO, SH. All authors read and approved the final manusrcipt.
